# The L-Rhamnose Biosynthetic Pathway in *Trichomonas vaginalis*: Identification and Characterization of UDP-D-Glucose 4,6-dehydratase

**DOI:** 10.3390/ijms232314587

**Published:** 2022-11-23

**Authors:** Matteo Gaglianone, Maria Elena Laugieri, Adriana Lucely Rojas, Maria Rosaria Coppola, Francesco Piacente, Pier Luigi Fiori, Michela Giulia Tonetti

**Affiliations:** 1 Department of Experimental Medicine, University of Genova, 16132 Genova, Italy; 2 Centro de Investigaciòn Cooperativa en Biociencias (CIC bioGUNE), 48160 Derio, Spain; 3 Department of Biomedical Sciences, University of Sassari, 07100 Sassari, Italy

**Keywords:** *Trichomonas vaginalis*, lipoglycan, L-rhamnose, UDP-D-glucose 4,6-dehydratase

## Abstract

*Trichomonas vaginalis* is the causative agent of one of the most widespread sexually transmitted diseases in the world. The adhesion of the parasite to the vaginal epithelial cells is mediated by specific proteins and by a complex glycan structure, the lipoglycan (TvLG), which covers the pathogen surface. L-rhamnose is an important component of TvLG, comprising up to 40% of the monosaccharides. Thus, the inhibition of its production could lead to a severe alteration in the TvLG structure, making the L-rhamnose biosynthetic pathway an attractive pharmacologic target. We report the identification and characterization of the first committed and limiting step of the L-rhamnose biosynthetic pathway, UDP-D-glucose 4,6-dehydratase (UGD, EC 4.2.1.76). The enzyme shows a strong preference for UDP-D-glucose compared to dTDP-D-glucose; we propose that the mechanism underlying the higher affinity for the UDP-bound substrate is mediated by the differential recognition of ribose versus the deoxyribose of the nucleotide moiety. The identification of the enzymes responsible for the following steps of the L-rhamnose pathway (epimerization and reduction) was more elusive. However, sequence analyses suggest that in *T. vaginalis* L-rhamnose synthesis proceeds through a mechanism different from the typical eukaryotic pathways, displaying intermediate features between the eukaryotic and prokaryotic pathways and involving separate enzymes for the epimerase and reductase activities, as observed in bacteria. Altogether, these results form the basis for a better understanding of the formation of the complex glycan structures on TvLG and the possible use of L-rhamnose biosynthetic enzymes for the development of selective inhibitors.

## 1. Introduction

*Trichomonas vaginalis* (Tv) is a flagellated parasitic protozoan; it represents the most common nonviral agent in sexually transmitted diseases in the world [1,2]. Tv can cause low weight at birth and prematurity during pregnancy and induces an augmented risk of cervical and prostate cancer [1,2,3]. The global prevalence in 2016 was estimated to be 5.5% for women and 0.6% for men: the total estimated incidence was 156.0 million trichomoniasis cases, with about 97% of cases occurring in developing countries with limited access to health care, where Tv has been associated with an increased risk of HIV infection [4]. Important health disparities also persist in the US and European countries, where a higher Tv incidence is correlated with ethnicity and socioeconomic status [2]. Metronidazole and related molecules are highly effective drugs that are currently used for therapy [2]; however, persistent and recurrent infections are now observed due to the increased presence of antimicrobial insensitive Tv strains, comprising 4% to 10% of infections [5]. Resistance to nitroimidazole-based molecules is of great concern, since few alternatives to the standard therapy exist; as a consequence, as now occurring for antibiotic-resistant bacteria, new targets for drug development are required.

The adhesion of the protozoan to the host epithelial tissues is mediated by specific proteins and by a large glycolipid structure, the lipoglycan (TvLG), previously indicated as lipophosphoglycan (LPG) [6,7]. TvLG mutants display reduced adherence and cytotoxicity to human ectocervical cells [7,8]. Moreover, the TvLG triggers an inflammatory response, leading to a selective cytokine upregulation in female reproductive tract epithelial cells, and it is also implicated in evasion from the host innate immunity [9,10,11,12].

The exact structure of TvLG is still debated; it differs markedly from the *Leishmania* and *Entamoeba* LPGs since it is devoid of the phosphosaccharide repeats [13]. It is anchored to the cell surface by an inositol–ceramide structure, and it is composed of L-rhamnose (L-Rha), N-acetyl-D-galactosamine, D-galactose, N-acetyl-D-glucosamine, D-glucose (D-Glc), and D-xylose. L-Rha forms an α1,3-linked polymeric backbone, from which lateral poly-lactosamine and poly-lacto-N-biose branches depart [13]. Depending on the clinical isolates, the L-Rha content ranges from 20% to 42% of the total monosaccharides, thus representing a substantial component of the Tv surface glycans [12]. Accordingly, due to its important role in TvLG formation, L-Rha metabolism represents an attractive target for a therapeutic intervention. However, the pathways and mechanisms of complex glycan formation in Tv are still poorly understood.

**Figure 1:**
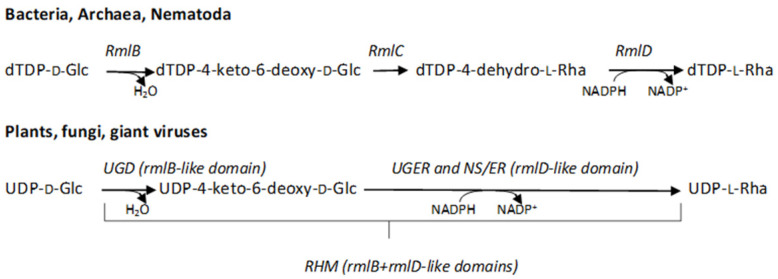
The L-Rha biosynthetic pathways in the different kingdoms. In Bacteria, Archaea, and Nematoda, the pathway starts from dTDP-D-Glc and proceeds through three steps, 4,6-dehydration, 3,5-epimerization, and 4-reduction, catalyzed by RmlB, RmlC, and RmlD, respectively. In Eukaryota and giant viruses, the preferred substrate of 4,6-dehydratase, which presents an *rmlB*-like domain, is UDP-D-Glc; this reaction is followed by epimerization and reduction carried out by single bifunctional enzymes characterized by an *rmlD*-like domain (L780/UGER from Mimivirus, NRS/ER from plants and fungi). In plants, the *rmlB*- and *rmlD*-like domains are also found in a single polypeptide chain (RHM).

L-Rha biosynthetic pathways have been described in organisms from all life domains and, surprisingly, in some giant viruses. In Bacteria and Archaea, the pathways start from dTDP-D-glucose (dTDP-D-Glc): after a first dehydration step, catalyzed by dTDP-D-Glc 4,6 dehydratase (RmlB) [14,15,16], two separate enzymes, a 3,5-epimerase (RmlC) [17,18,19] and an NADPH-dependent 4-keto-reductase (RmlD) [20], lead to the formation of dTDP-L-Rha (Figure 1). A similar mechanism involving separate epimerase and reductase activities and a preference for the dTDP-bound substrate has also recently been reported for the L-Rha pathway in *Caenorhabditis elegans* [21]. On the other hand, in fungi and viruses the preferred initial substrate is UDP-D-glucose (UDP-D-Glc); moreover, after the initial dehydration catalyzed by proteins with an *rmlB*-like domain (UGD), the epimerization and the following reduction are catalyzed by bifunctional enzymes characterized by a single *rmlD*-like domain (UGER) (Figure 1) [22,23]. In plants, the NRS/ER proteins with a single epimerase/reductase *rmlD*-like domain were found [24], as observed in fungi and giant viruses. Moreover, in plants the RHM enzymes comprise both the *rmlB*- and *rmlD*-like domains fused together, and again the preferential substrate is UDP-D-Glc [25]. Thus, in most eukaryotes distinct epimerases containing the cupin *rmlC*-like domain are not required. However, a recent report from Wagstaff et al. demonstrated that some Haptophytes and red algae, unlike Viridiplantae, use enzymes containing both the *rmlC*- and *rmlD*-like domains to catalyze the last steps of the pathway, showing a preference for the dTDP-sugars as substrates [26].

The aim of this study was the identification of the L-Rha biosynthetic pathway in Tv in order to contribute to the understanding of the mechanisms leading to complex glycan formation in this pathogen as possible targets for new therapeutic developments. We have identified and characterized the first enzyme of the pathway, UDP-D-Glc 4,6-dehydratase (UGD, EC 4.2.1.76). Moreover, based on the sequence analysis of the enzymes possibly involved in the following synthetic steps, we propose that the Tv L-rhamnose pathway presents properties that are intermediate between the eukaryotic and the prokaryotic pathways. Specifically, it uses the UDP-bound sugar as a preferential substrate, as in eukaryotes, but it requires separate enzymes to change the sugar configuration and to carry out the final reduction step, as observed in prokaryotes.

## 2. Results

### 2.1. Identification of the Genes Involved in the L-Rha Pathway in Tv

Several sequences in the Tv genome have been already annotated as putative proteins involved in the L-Rha biosynthetic pathway. In this study, we initially performed a comprehensive BLAST analysis of the Tv genome and at least seven sequences were identified, which displayed significant similarity to the already characterized bacterial and eukaryotic enzymes. These sequences, containing *rmlB*-, *rmlC*-, and *rmlD*-like domains, and their most important features are reported in Table 1, which also indicates the identified orthologs in *Tritrichomonas foetus*, a pathogen closely related to Tv; *T. foetus* infects cattle, and it exposes L-Rha in the LG [27]. The presence of multiple related sequences possibly involved in the Tv L-Rha pathway is not surprising, since it is well known that the Tv genome is characterized by extensive gene duplication and by the presence of pseudogenes [28,29,30]. An analysis of EST abundance using the TrichoDB database (https://trichdb.org, accessed on 4 april 2022) indicated that all the Tv genes reported in Table 1 were expressed.

TVAG_414560 was the only putative protein containing an *rmlB*-like domain, identifying it as a UDP/dTDP-D-Glc 4,6 dehydratase (UGD). The best BLASTp hits were with proteins from several protists, including *T. foetus* (77% identity, Table 1); *Histomonas meleagridis*, another member of the Trichomonida (70% id.); *Sphaeroforma artica* (59% id.); *Thecomonas trahens* (58% id.); and *Entamoeba* species (55% id). Significant similarity (about 50% id.) was also observed with the N-terminal domain of the plant RHM proteins and with proteins of unknown function in higher metazoans (47% id. with human TGDS). The alignment with homologs from representative organisms is reported in Supplementary Figure S1, which also highlights the residues involved in catalysis. The data from EST sequencing from TrichoDB and RNAseq experiments suggest high levels of expression, which can be modified by culture conditions; for instance, a six-fold upregulation was reported by RNASeq during glucose deprivation [31], while downregulation occurred following tetracycline treatment [32]. The significance of these findings is not currently clear, but they suggest a central role of this gene in pathway regulation.

A more complex situation was found for the following steps of the L-Rha pathway. TVAG_357160 is annotated as dTDP-4-dehydrorhamnose reductase and it is characterized by an *rmlD*-like domain, albeit displaying a low score with plant NRS/ER, the C-terminal domain of RHM enzymes and Mimivirus L780, whose epimerase/reductase activity was confirmed [23,24]. The BLAST analysis revealed the highest similarity (about 46% id. on the whole sequence) with putative dTDP-4-dehydro-L-rhamnose reductases from *Bacteroides* and *Prevotella* species. An ortholog of TVAG_357160 was not identified in *T. foetus* (Table 1) and *H. meleagridis*. The alignment of TVAG_357160 with representative sequences is reported in Supplementary Figure S2, which highlights the residues identified for the catalytic mechanism in this class of enzymes. RNAseq experiments suggested very low levels of expression for this gene [31,32].

Two other sequences, TVAG_313960 and TVAG_340730, are annotated as dTDP-4-dihydrorhamnose 3,5-epimerase, and they are characterized by the presence of a single cupin *rmlC*-like domain of about 180 residues, followed by long C-terminal regions with no significant similarity between them and with proteins present in the databases (Table 1). These proteins are indicated from here on as TvRmlC proteins. The identity between the two cupin domains was about 50%, and the best BLASTp hits were uncharacterized proteins from unicellular eukaryotes, the N-terminal cupin-like region of the RmlCD enzyme from Haptophyta [26], and hypothetical sequences from some lower metazoans. Identity with the well-characterized 3,5-epimerases involved in L-Rha synthesis in Bacteria was around 34–37%, while it was 39% with the *C. elegans* RML-3 enzyme [21]. The sequence alignment, reported in Supplementary Figure S3, indicates that the RmlC residues essential for the catalytic activity are well conserved [17,18,19]. However, a region at the N-terminus involved in the formation of the dimer interface was not present in the trichomonad proteins.

Other sequences, TVAG_463120, TVAG_016600, and TVAG_401850, contain both the typical cupin *rmlC*-like and the *rmlD*-like domains (indicated from here on as TvRmlCD proteins), possibly derived by a gene fusion event (Table 1). TVAG_016600 and TVAG_401850, derived by a recent gene duplication, displayed 98% identity, while TVAG_463120 shared about 46% identity with them. TVAG_463120 and 401850 are wrongly annotated in the databases as dTDP-glucose 4,6-dehydratases. All three putative proteins share about 50–60% identity with a single gene from *T. foetus* (TRFO_30760, Table 1) and *H. meleagridis*. The *rmlD*-like C-terminal domains displayed moderate similarity with plant NRS/ER enzymes and Mimivirus L780 (about 30% id.) [23,24]. However, the putative Tv proteins did not contain the Lys (Lys293 in TVAG_463120) belonging to the Tyr-Xxx-Xxx-Lys motif, which is very well conserved in the active site for SDR enzymes (Supplementary Figure S4) [33]; instead, the Lys was substituted with an Arg. Besides Trichomonadida, the best hits for the N-terminal cupin domain of the TvRmlCD proteins were with bacterial sequences annotated as dTDP-D-dehydrorhamnose 3,5-epimerase. However, unlike TVAG_313960 and TVAG_340730, the residues required for catalytic activity were not conserved, as evidenced by the sequence alignment in Supplementary Figure S5. Moreover, a low similarity was observed with the RmlCD bifunctional enzymes from Haptophyta [26].

Altogether, the sequence analyses suggest the presence of a nonconventional eukaryotic pathway for L-Rha production in Tv, reminiscent of the bacterial and *C. elegans* mechanism in which epimerization and reduction are performed by distinct proteins [21] and of the Haptophyta mechanism in which the *rmlC*- and *rmlD*-like domains are fused in a single polypeptide chain [26].

### 2.2. Production of the Recombinant Proteins and Identification of the Enzymatic Activities

To determine the possible roles of these identified sequences in the NDP-L-Rha pathway, all the proteins, with the exception of TVAG_016600, were produced in *E. coli* as GST-fusion proteins using the pGEX-6P1 vector. After GST removal, all the proteins could be obtained in a soluble form and in good yield and purity (Supplementary Figure S6).

**Figure 2:**
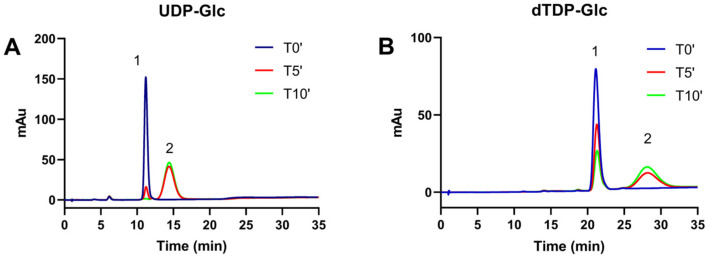
Anion-exchange HPLC analysis of TvUGD products. Incubations were performed in identical conditions at 25 °C using 0.3 mM substrates; samples were withdrawn at different time points and analyzed by anion-exchange chromatography. The progressive formation of new peaks was observed for both UDP-D-Glc (Panel (**A**)) and dTDP-D-Glc (Panel (**B**)). The complete conversion of UDP-D-Glc to the product was completed in less than 10 min, whereas dTDP-D-Glc was still present at that time, indicating a lower activity of the enzyme for this substrate. Peak 1: substrate, NDP-D-glucose. Peak 2: product, NDP-4-keto-6-deoxy-D-glucose.

TVAG_414560, the putative dehydratase, was initially tested using UDP-D-Glc and dTDP-D-Glc as substrates using an HPLC-based method. As shown in the chromatograms of Figure 2A,B, the incubation of both substrates with the recombinant enzyme led to the progressive appearance of a new peak with a longer retention time, corresponding to the dehydration products, as also confirmed using the Mimivirus UGD product as a reference [23]. When analyzed in identical conditions, the activity was significantly higher for UDP-D-Glc as a substrate compared to dTDP-D-Glc. The product identity was confirmed by an ESI/MS analysis of the reaction mixture after the conversion of UDP-D-Glc into the product: two main species were found, one at m/z 547.17, corresponding to UDP-4-keto-6-deoxy-D-Glc, and a second at m/z 565.17 for the substrate (Supplementary Figure S7A). These data confirm the identification of TVAG_414560 as UDP-D-Glc 4,6 dehydratase (TvUGD, EC 4.2.1.76).

**Figure 3:**
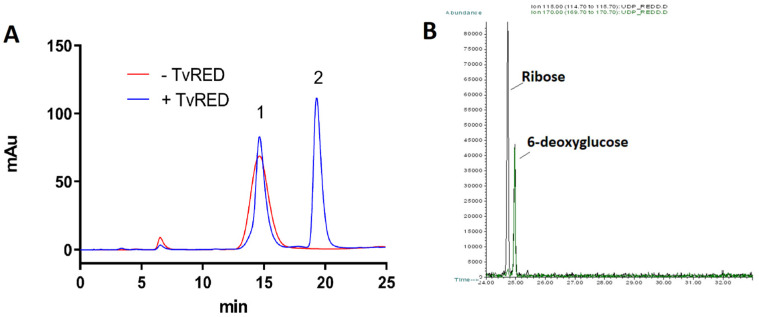
Analysis of TvRED products. (**A**) Anion-exchange HPLC analysis. UDP-4-keto-6-deoxy-D-Glc (red chromatogram) was produced using TvUGD and incubated with TvRED in the presence of NADPH (blue chromatogram). A change in the substrate peak shape (peak 1, red line: UDP-4-keto-6-deoxy-D-glucose; blue line: UDP-6-deoxy-D-glucose) with parallel NADP^+^ formation (peak 2, blue line) was observed, suggestive of a reductase reaction. In this chromatographic condition, NADPH was retained in the column. (**B**) Alditol acetate GC-MS analysis TvRED product. The reaction product was isolated, subjected to acid hydrolysis, and converted to alditol acetates. The chromatograms were obtained by the extraction of m/z 115 (black line) and m/z 170 (green line) ion currents; for clarity, the chromatogram between 24 and 33 min is displayed. The analysis revealed the presence of ribose, derived from the UDP moiety, and 6-deoxyglucose. The identification of the monosaccharides was achieved by a comparison with the retention times of sugar standards and an analysis of the fragmentation spectra.

The TvUGD product was then used to test the activity of the other recombinant proteins, using them either alone or in different combinations. The incubation of UDP-4-keto-6-deoxy-D-Glc with TVAG_357160 in the presence of NADPH induced a modification in the shape of the substrate peak and the appearance of a new peak corresponding to NADP^+^ (Figure 3A). The formation of NADP^+^ in the chromatogram and the disappearance of NADPH, monitored by absorbance at 340 nm (not shown), are highly suggestive of a reductase activity (TvRED). This activity was also confirmed by ESI-MS, which reported an m/z of 549.17, consistent with the presence of a UDP-6-deoxyhexose (Supplementary Figure S7B). However, the new peak did not display a retention time corresponding to the UDP-L-Rha standard. To conclusively demonstrate TvRED activity, the purified product was subjected to a GC-MS analysis which showed the presence of 6-deoxyglucose together with the ribose from the nucleotide moiety (Figure 3B). These findings clearly indicate that TvRED does not have 3,5-epimerase activity and it only possesses a stereospecific reductase activity on the substrate 4-keto group.

In order to identify the 3,5-epimerase or other possible epimerase/reductases, incubations were performed on the TvUGD products or, as alternative using NDP-D-Glc in the presence of TvUGD, using different combinations and amounts of all the purified TvRED, TvRmlC, and TvRmlCD recombinant proteins. Epimerase and/or reductase activities were not detected in all the conditions tested for TvRmlC and TvRmlCD proteins. A 4-keto reductase activity was observed only after TvRED addition to the enzyme mix; however, despite the presence of the putative epimerases, UDP-6-deoxyglucose was always detected as the final product (not shown).

### 2.3. TvUGD Kinetic Properties and Quaternary Structure Characterization

Incubations of TvUGD performed using different UDP-D-Glc concentrations followed by HPLC analysis revealed a K_m_ of 21.3 ± 3.4 µM and a k_cat_ of 0.39 ± 0.01 s^−1^ for this substrate. On the other hand, the K_m_ for dTDP-D-Glc was about 362.8 ± 73.4 µM, about twenty-fold higher compared to UDP-D-Glc, with a kcat of 0.36 ± 0.03 s^−1^, thus demonstrating a higher affinity of TvUGD for the UDP-bound substrate. These findings are comparable with the enzymatic properties previously obtained in our laboratory using the viral UGDs [26].

The optimal pH for TvUGD activity is around 7.0, showing a particularly sharp decrease in basic conditions (pH higher than 8.5) while maintaining good activity until pH 5.0. The enzyme is not affected by the addition of bivalent cations and NAD(P)^+^, indicating that the coenzyme remains strictly bound to the protein during purification. When kept concentrated (at least 2 mg/mL) at 4 °C, TvGD was proven to be stable, with a negligible loss of activity for up to two weeks. Upon the preincubation of the enzyme (1.5 mg/mL) for 45 min at 25 °C, 30 °C, and 37 °C, the residual activities were about 90%, 70%, and 40%, respectively, compared to the protein kept at 4 °C. Indeed, TvUGD, due to its high catalytic efficiency and good stability, proved to be very efficient in applications for an in vitro UDP-L-Rha-generating system [34].

The elution time of the purified protein in size-exclusion chromatography allowed us to calculate a molecular weight of around 91 kDa, suggestive of a dimer, in agreement with the oligomeric assembly of the previously reported NDP-D-Glc 4,6-dehydratases [14,15,16,26].

### 2.4. TvUGD Modeling

**Figure 4:**
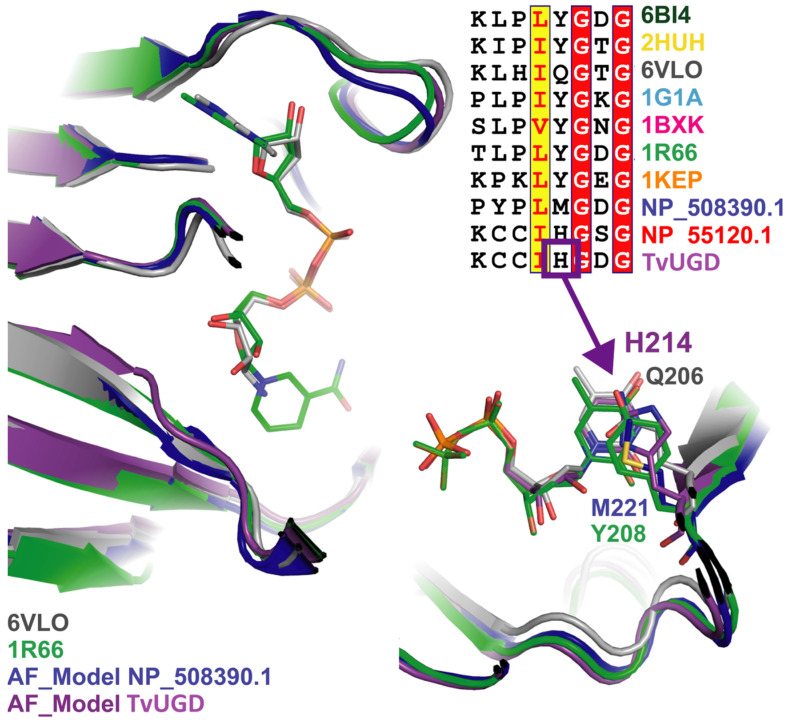
Close-up view of the 3D alignment of the NDP-D-Glc 4,6-dehydratase active site. The residues in the same position of TvUGD His214 are shown in sticks; as Tyr is conserved in most of the structures, only the one from PDB:1R66 is shown. The inset represents a portion of sequence alignment comprising TvUG His214. *Acanthamoeba polyphaga* Mimivirus R141 (PDB:6VLO in grey), *Salmonella enterica* (PDB:1G1A in cyan), *Escherichia coli* (PDB:1BXK in magenta), *Streptomyces venezuelae* (PDB:1R66 in green), *Streptococcus suis* (PDB:1KEP in orange), *Homo sapiens*, NP_508390 (AlphaFold2 model in blue), CELE_F53B1.4 from *Caenorhabditis elegans*, NP_055120 (AlphaFold2 model in red), model of TvUGD, TVAG_414560 (Alphafold2 model in violet).

Since the kinetic results indicated a clear preference for the UDP-bound substrate, we investigated the structural determinants of nucleotide recognition by TvUGD using Alpha-fold modeling [35]. The alignment and 3D superposition of TvUGD homologous proteins confirmed that this enzyme is closely related to those that perform the same reaction from Bacteria, Archaea, Mimivirus, and Eukarya (Supplementary Figure S8). However, some differences were observed in the binding site of the substrate. In particular, *Streptomyces venezuelae* (PDB:1R66) Tyr208 (and the corresponding Tyr residues from other prokaryotic enzymes) develops a π-stacking interaction with the thymidine ring [14,15,16]. Sequence alignments indicate that the corresponding position is occupied by a His residue in TvUGD (His214) and other eukaryotes, Gln206 in Mimivirus, and Met221 in the *C. elegans* enzyme (Figure 4).

**Figure 5:**
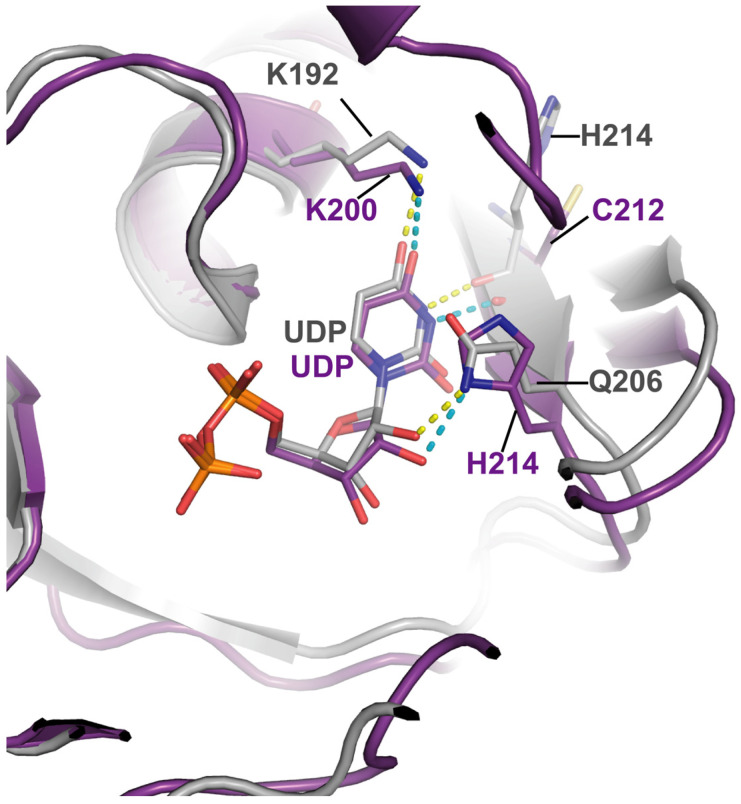
Close-up view of active site models of TvUGD (in violet) and of Mimivirus R141 (PDB:6VLO, in grey). Dashed lines indicate possible hydrogen bond interactions with the side chain of His214 and Gln206 with the C-2′ hydroxyl group.

The presence of His214 in the active site of TvUGD allows the π- stacking of its imidazole group with the pyrimidine ring of UDP-D-glucose and dTDP-D-Glc, which is consistent with the ability to use both substrates. However, the interaction of the ND1 of the imidazole group with the C-2′ hydroxyl group of the ribose moiety of UDP (Figure 5) suggests a preference for UDP-D-Glc, in agreement with our observation that this enzyme has a twenty-fold higher affinity for this substrate compared to dTDP-D-Glc. Similarly, modeling of the UDP in the Mimivirus UGD active site suggests that the Gln206 lateral chain can also develop the same type of interaction with the C-2′ hydroxyl group (Figure 5), suggesting again a preference for the UDP-bound substrate for this enzyme, as indicated by previous results obtained in our laboratory [23].

### 2.5. Phylogenetic Analysis of TvUGD

**Figure 6:**
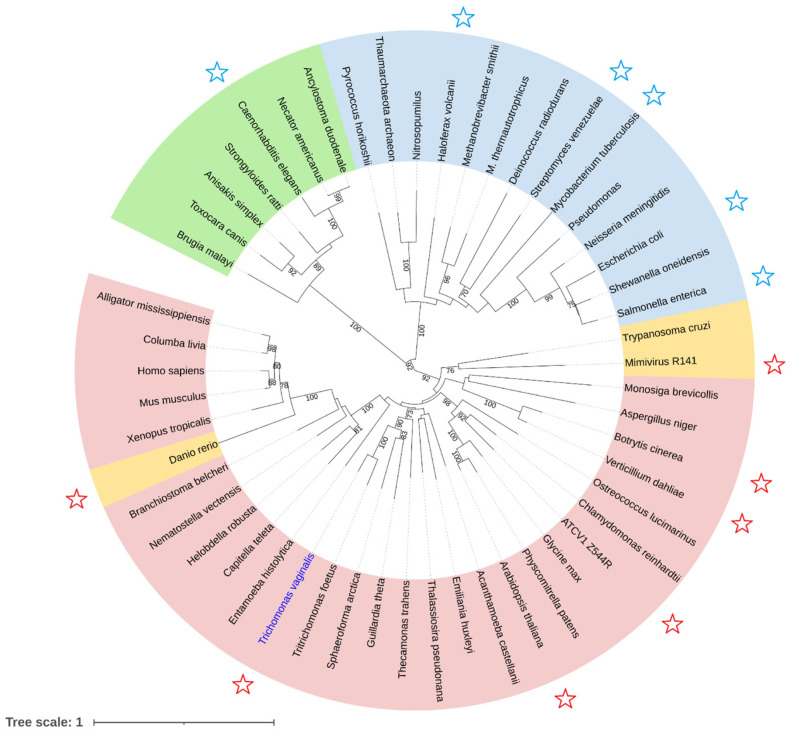
Phylogenetic analysis of representative NDP-D-Glc 4,6-dehydratases from different taxa. Sequences displaying specific residues in the position corresponding to TvUGD His214 are indicated by different colors: Met, green; Tyr, blue; Gln, orange; His, red. Blue stars indicate characterized enzymes that use dTDP-D-Glc, while red stars are used for the proteins showing a clear preference for the UDP-bound substrate. Details of the sequences used for analysis are reported in Supplementary Table S1. TvUGD is highlighted in blue.

The sequence alignments of TvUGD and orthologs from other taxa showed a remarkably high conservation of NDP-D-Glc 4,6-dehydratases across all domains of life, including some lower metazoans and Vertebrata. A phylogenetic analysis using representative sequences from different taxa was then performed (Figure 6). Several clades emerged from the phylogenetic analysis. Two sister clades comprise all dehydratases reported so far that use dTDP-D-Glc as a preferred substrate (indicated by a blue star) [14,15,16,21]: one includes the bacterial and archaeal sequences (highlighted in light blue), which are characterized by the presence of the very well conserved Tyr residue, and the other includes the nematode sequences (in green), all displaying Met in the corresponding position. The clustering of nematode dehydratase with the bacterial enzymes as well as the use of separate epimerase and reductase enzymes, as it occurs in bacteria, are suggestive of an independent acquisition of the L-Rha pathway in roundworms that is not evolutionarily related to the eukaryotic pathway.

TvUGD clusters in another clade, indicated in light red, which includes several subclades containing the eukaryotic (plants, fungi, and Metazoa) and giant virus enzymes. All sequences herein contain the His residue, as TvUGD and, accordingly, the already characterized enzymes display a clear preference for UDP-D-Glc (indicated by a red star) [22,23,25]. A further subclade including the Mimivirus and Kinetoplastidia enzymes is highlighted in orange and contains few members presenting Gln in the corresponding position. Gln is also found in zebrafish (*Danio rerio*), whose sequence is quite divergent from the other members of Vertebrata (Figure 6). However, the results recently obtained in our laboratory indicated that the zebrafish enzyme is able to catalyze the NDP-D-Glc dehydration and confirmed a higher affinity for the UDP-bound substrate (Tonetti et al., unpublished results). Collectively, the sequence analysis and phylogenetic results give further support to the proposed mechanism underlying the preferential recognition of ribose vs. deoxyribose in TvUGD mediated by His214.

## 3. Discussion

L-Rha is an important component of the TvLG covering the parasite surface: a poly-rhamnose backbone supports poly-LacNAc and poly-Lacto-N-biose lateral chains, which are involved in adhesion to host epithelial cells, making the L-Rha biosynthetic pathway an attractive pharmacologic target [12]. In this study, we have identified and characterized the first enzyme of the Tv L-Rha pathway, TvUGD, which catalyzes the dehydration of UDP-D-Glc to form UDP-4-keto-6-deoxy-D-Glc. On the other hand, the identification of the enzymes responsible for the second part of the Tv L-Rha pathway proved to be elusive, and the enzymatic activity of the expressed recombinant proteins could not be demonstrated, with the exception of a high 4-keto reductase activity for TVAG_357160. However, this enzyme does not display the associated 3,5-epimerase activity, which is found in other eukaryotes and Mimivirus proteins [22,23,24]. We cannot definitively conclude that this protein has a key role in the last reduction step since it is not found in the closely related *T. foetus*, which is expected to have a functional L-Rha pathway [27], and in *H. meleagridis*, a related trichomonad parasite that causes histomonosis in poultry [36]. The high similarity of TvRED (TVAG_357160) to 4-keto reductases from Bacteroidetes, which are often present as commensal organisms in the gut and vaginal microbiota [11,37], suggests the possibility of its acquisition after the radiation of the trichomonad lineage through a recent LGT, which is known to be an important event shaping the Tv genome [38].

We were not able to demonstrate epimerase activity for the TvRmlC proteins (TVAG_340730 and TVAG_313960); however, the presence of all the residues involved in the catalytic mechanism in the *rmlC*-like domain suggests that they are active enzymes related to the bacterial and archaeal enzymes with a role in the L-Rha pathway. The presence of distinct proteins for epimerization and reduction is typically found in prokaryotes; however, this strategy was also identified in few eukaryotes, such as *C. elegans* and Haptophytes [21,26]. In particular, in *Prymnesium parvum* the *rmlC*- and rmlD-like domains are fused in a single polypeptide chain, as also occurs in TvRmlCD proteins (TVAG_413120, TVAG_016600, and TVAG_401850). However, the lack of the residues involved in catalysis in the characterized 3,5-epimerases in the *rmlC*-like domain of TvRmlCD poses questions about their role in the reaction. On the other hand, the residues recognized to be essential for the catalytic activity and substrate recognition are conserved in the *rmlD*-like domain of TvRmlCD, with the exception of the substitution of the Lys of the catalytic triad with an Arg residue.

The incubation of the purified proteins together in different combinations did not provide successful results. However, an explanation could be that, in order to be functional, proteins need to be expressed together in *E. coli* to form complexes, as already reported for the L-Rha pathway of C. elegans [21]. In fact, besides RML-1 (the dTDP-D-Glc pyrophosphorylase) and RML-2 (dTDP-D-Glc 4,6-dehydratase), this pathway proceeds with RML-3 (3,5-epimerase) and RML-4 (4-keto-reductase). Interestingly, to obtain a functional recombinant RML-4 in *E. coli*, an accessory protein, RML-5, needs to be coexpressed. RML-5 is composed of two domains, an N-terminal *rmlB*-like domain and a C-terminal *rmlD*-like domain, a structure reminiscent of the plant RMH enzymes; however, some of the residues essential for catalysis are not conserved in RML-5, suggesting a scaffolding role for this protein. The coexpression of the Tv proteins in *E. coli* is made difficult by the presence of several isoforms possibly involved in the L-Rha pathway, which could also comprise pseudogenes. For the reasons reported above, a more suitable organism to study the enzymatic activities for the second part of the pathway is *T. foetus*, which contains L-Rha in LG [27] and encodes single orthologs for each Tv gene (Table 1). Single orthologs for all Tv proteins are also found in *H. meleagridis*. Further studies with these organisms can be addressed to clarify this issue using the recently developed knock-out strategies [39].

The TvUGD protein displays kinetic properties similar to the reported UDP-D-Glc 4,6-dehydratases previously characterized in our laboratory [23]. As observed for other eukaryotic and viral dehydratases, TvUGD can use both the UDP- and dTDP-bound sugar substrates, but it shows a clear preference for UDP-D-Glc, unlike the bacterial and archaeal dehydratases, which show a strict preference for the dTDP-bound substrate [14,15,16]. Using modeling and sequence analysis, we propose that the nucleotide preference is not determined by the pyrimidine ring but rather by the presence of the ribose moiety vs. deoxyribose; specifically, the possibility of His214 to form hydrogen bonds with both the C2′- and C3′- hydroxyl groups of ribose confers more stability to substrate binding. The importance of this His residue is also highlighted by the very good conservation in eukaryotic enzymes, which show a strong preference for UDP-D-Glc. Moreover, the modeling of UDP in the Mimivirus R141 3D structure suggested that the Gln side chain can also form interactions with both ribose C2′- and C3′- hydroxyl groups, thus supporting a higher affinity for the UDP-bound substrate. Indeed, this hypothesis is consistent with our previously reported analyses on Mimivirus R141, which indicated that the viral enzyme also uses UDP-D-Glc as a preferential substrate [23]. Recently, Ferek et al. reported an identical affinity for both UDP-D-Glc and dTDP-D-Glc for Mimivirus R141 [40]; the reasons are not clear, and further analyses could clarify this issue.

Understanding the determinants of substrate binding for the dehydratases involved in L-Rha production is also important for practical applications since this pathway has been proposed as a target for the development of new drugs to affect both the prokaryotic and eukaryotic enzymes. Indeed, L-Rha is essential for the infectivity and virulence of several pathogens, including *Mycobacterium*, *Streptococcus*, and *Candida* species [41,42], microorganisms that are all showing increasing resistance to the antibiotics currently in use. Preliminary data also make this pathway a possible attractive target for roundworm infestations [21]. Several candidates have already been proposed for lead discovery in the treatment of bacterial infections as inhibitors of different enzymes of the pathway, including the dehydratases [43,44,45]. The identification of TvUGD now opens the possibility to also evaluate these new compounds in Tv. However, a drawback of the use of dehydratase inhibitors for therapeutic intervention could be the presence of close homologs of the NDP-D-Glc 4,6-dehydratases in higher metazoans, including Vertebrata. The function of these TvUGD orthologs is presently unknown since a functional L-Rha biosynthetic pathway was never described in these organisms; however, recent results obtained in our laboratory demonstrate that the zebrafish TGDS (TDP-glucose 4,6-dehydratase) is able to catalyze UDP-D-glucose dehydration (Tonetti et al., unpublished results). Mutations in human TGDS have been implicated in Catel–Manzke syndrome, a rare autosomal recessive genetic disease characterized by hyperphalangism and clinodactyly, facial dysmorphism with hypognathia and cleft palate (Pierre Robin sequence), other associated skeletal alterations and heart malformations [46]. The pathogenic mechanisms associated with Catel–Manzke syndrome are still unknown. Thus, in the perspective to use NDP-D-Glc 4,6-dehydratases as pharmacologic targets, further studies will also be required to understand the role of TGDS in human cells.

## 4. Materials and Methods

### 4.1. Sequence Identification and Analysis

The putative sequences that are possibly involved in the L-rhamnose biosynthetic pathway were identified in the Tv genome using blastp and tblastn (https://blast.ncbi.nlm.nih.gov/Blast.cgi, lastly accessed on 28 July 2022) on the nr protein sequences and nucleotide collection using several identified dehydratases, epimerases, and reductases from eukaryotes and prokaryotes. Sequence alignments were performed using M-Coffee on the T-coffee server (http://tcoffee.crg.cat/ lastly accessed on 10 August 2022). Figures were produced using ESPript (https://espript.ibcp.fr/ESPript/ESPript/ lastly accessed on 10 August 2022) [47].

### 4.2. Cloning and Expression of the Recombinant Proteins in E. coli

Tv cDNA was obtained from reference strain G3 and tested by a specific PCR to confirm the absence of *Mycoplasma hominis* endosymbionts; after amplification using Pfu polymerase (NEB) and sequence-specific primers, the PCR products were cloned in pGEX-6-P1 (GE Healthcare) using restriction cloning. The primers used for amplifications are reported in Supplementary Table S2, which highlights the restriction enzymes used (all from NEB). The sequencing of the vector containing the correct insert was performed by TibMolBiol (Genova, Italy). Protein expression was obtained using BL21 *E. coli* cells and purified on GSH-sepharose 4B (GE Healthcare, Chicago, IL, USA), as described in [23]. The recovery of the native proteins from the GST-fusion protein was achieved after precision protease cleavage (GE Healthcare) and concentration using an Amicon Ultra-4 filter (Millipore, Burlington, MA, USA).

The protein concentrations were determined by UV spectrophotometry at 280 nm using a calculated extinction coefficient with the Protparam tool on the Expasy server [48]; purity was determined by SDS-PAGE. The quaternary structure was determined by size-exclusion chromatography using a TSK-gel 3000 column, as described in [23].

### 4.3. Analysis of the Enzymatic Activities

The TvUGD activity was tested using UDP-D-glucose (Merck, Rahway, NJ, USA) and dTDP-D-glucose. This latter compound was produced in our laboratory using glucose-1P and dTTP (both from Merck) as a substrate and recombinant *E. coli* dTDP-D-glucose pyrophosphorylase in the presence of inorganic pyrophosphatase (Merck), as previously described [49]; this allowed the complete conversion of the substrates in the product dTDP-D-Glc. This was purified using SPE, and its identity and purity were confirmed by ion-exchange HPLC and ESI-MS, as reported in [23,50]. The conversion of the nucleotide sugar substrates to the intermediate compound NDP-4-keto-6-deoxy-D-glucose was determined by ion-exchange HPLC [23] at different time points of incubation in order to reliably measure V_0_. Due to the wide shape of the product peak, the conversion was determined by analyzing the decrease in the area under the peak for the substrates compared to the standards at different concentrations; the areas of the standard peaks increased linearly in the concentration range from 6.25 µM to 1 mM (r^2^ > 0.99). The identity of the NDP-4-keto-6-deoxy intermediate products was confirmed by ESI-MS, as reported in [23,50]. The standard activity assay for TvUGD was performed in 50 mM Tris/HCl and 150 mM NaCl at pH 7.0 and 25 °C using different concentrations of UDP-D-glucose, dTDP-D-glucose, and enzyme, with or without the addition of bivalent ions and NAD(P)^+^; the steady-state kinetic parameters were determined by fitting the experimental data to the Michaelis–Menten equation using Prism Graphpad 7. The K_cat_ (1/s) was calculated from the maximum initial velocity, k_cat_ = V_max_/E, where E is the total enzyme concentration (µmol/L), assuming a molecular mass of 39 kDa for the TvUGD monomer. The results were obtained from at least three independent protein preparations.

The optimal pH was determined using a phosphate buffer at different pHs (from 5.5 to 8.0) and Tris/HCl (from 7.0 to 9.5), with 200 µM UDP-D-glucose as a substrate at 25 °C. The thermal stability was analyzed after the preincubation of TvUGD (1.5 mg/mL) for 45 min at 4 °C, 25 °C, 30 °C, and 37 °C; the activity was then tested in standard conditions. The stability in long-term storage was determined by testing the activity in standard conditions after several weeks of storage of the concentrated protein (>2 mg/mL) at 4 °C.

To test the enzymatic activity of the other recombinant proteins possibly involved in the second half of the L-Rha pathway, UDP-D-Glc and dTDP-D-Glc were initially completely converted to their respective intermediate compounds by TvUGD. After heat inactivation at 80 °C for 3 min, the purified proteins were added, either alone or together, in different combinations and concentrations in the presence or absence of NAD(P)H and bivalent cations. At different time points, the samples were analyzed by HPLC and ESI-MS, as described above. The identity of the 6-deoxyhexose obtained after the NADPH-dependent reduction in the intermediate by TvRED was determined after the purification of the nucleotide sugar, acid hydrolysis, and alditol acetate GC-MS analysis, as described in [50]. To verify the possible presence of only epimerase activity, the samples were also treated with NaBH_4_ before acid hydrolysis in order to reduce the 4-keto group and were then subjected to acid hydrolysis, reduction, and alditol acetate GC-MS, as reported in [51].

### 4.4. Modeling of TvUGD in Complex with UDP

A model of the 3D structure of the TvUGD was obtained using Alphafold2 [35,52]. The position of the UDP was inferred by superposing this model with the structure of dTDP-D-glucose 4,6-dehydratase from *Acanthamoeba polyphaga* mimivirus (PDB:6VLO). Then, the orientation of the best rotamer of His214 was found using Coot [53], and 20 structure idealization cycles were performed with REFMAC [54]. In this way, a plausible model of the dehydratase in complex with UDP was obtained.

In the other hand, to produce the structural alignment, the models of the 3D structures of dTDP-D-glucose 4,6-dehydratase from *Homo sapiens* and CELE_F53B1.4 from *C. elegans* were taken from Alphafold2 database [52]. The alignment was performed using the DALI server [54,55].

### 4.5. Phylogenetic Analysis

Representative sequences from the different taxa were retrieved from the nr database using BLASTp and aligned using CLUSTALW. The evolutionary history was inferred using the neighbor-joining method [56]. The optimal tree with a total branch length = 18.39665041 is shown. The percentages of replicate trees in which the associated taxa clustered together in the bootstrap test (1000 replicates) are shown next to the branches [57]. The evolutionary distances were computed using the JTT matrix-based method [58] and are expressed in the units of the number of amino acid substitutions per site. The rate variation among sites was modeled with a gamma distribution (shape parameter = 1). This analysis involved 52 amino acid sequences. All positions with less than 95% site coverage were eliminated, i.e., fewer than 5% alignment gaps, missing data, and ambiguous bases were allowed at any position (partial deletion option). There was a total of 290 positions in the final dataset. Evolutionary analyses were conducted in MEGA X [59]. The tree was generated using iTOL (https://itol.embl.de/, accessed on 12 May 2022) [60].

## Figures and Tables

**Table 1:** Sequences identified in the T. vaginalis genome by BLAST analysis using enzymes of the NDP-L-rhamnose pathways in Bacteria, Eukaryota, and giant viruses. ND, not detected.

Gene Name	Length	Domain(s)	Pfam acc. n.	Predicted Activity	Ortholog in T. Foetus (% id.)
TVAG_414560	348 aa	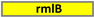	PF16363	4,6-dehydratase	TRFO_03667 (77%)
TVAG_357160	285 aa	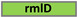	PF04321	4-keto-reductase	ND
TVAG_463120	450 aa	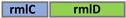	PF00908/PF04321	3,5-epi,4-keto-red	TRFO_30760 (59%)
TVAG_016600	449 aa	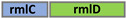	PF00908/PF04321	3,5-epi,4-keto-red	TRFO_30760 (59%)
TVAG_401850	448 aa	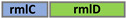	PF00908/PF04321	3,5-epi,4-keto-red	TRFO_30760 (51%)
TVAG_340730	347 aa	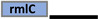	PF00908	3,5-epimerase	TRFO_42104 (37%)
TVAG_313960	420 aa	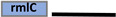	PF00908	3,5-epimerase	TRFO_42104 (42%)

## Data Availability

Not applicable.

## Supplementary Materials

The supporting information can be downloaded at: https://www.mdpi.com/article/10.3390/ijms232314587/s1.
