# Supplementary material for: The L-Rhamnose Biosynthetic Pathway in Trichomonas vaginalis: Identification and Characterization of UDP-D-Glucose 4,6-dehydratase
Source: Int J Mol Sci. 2022 Nov 23;23(23):14587. doi: 10.3390/ijms232314587 (PMC9741107; doi:10.3390/ijms232314587)
Supplement: Supplementary file 1 [file ijms-23-14587-s001.zip › ijms-2043317-supplementary.pdf]

Supplemental information

Figure S1

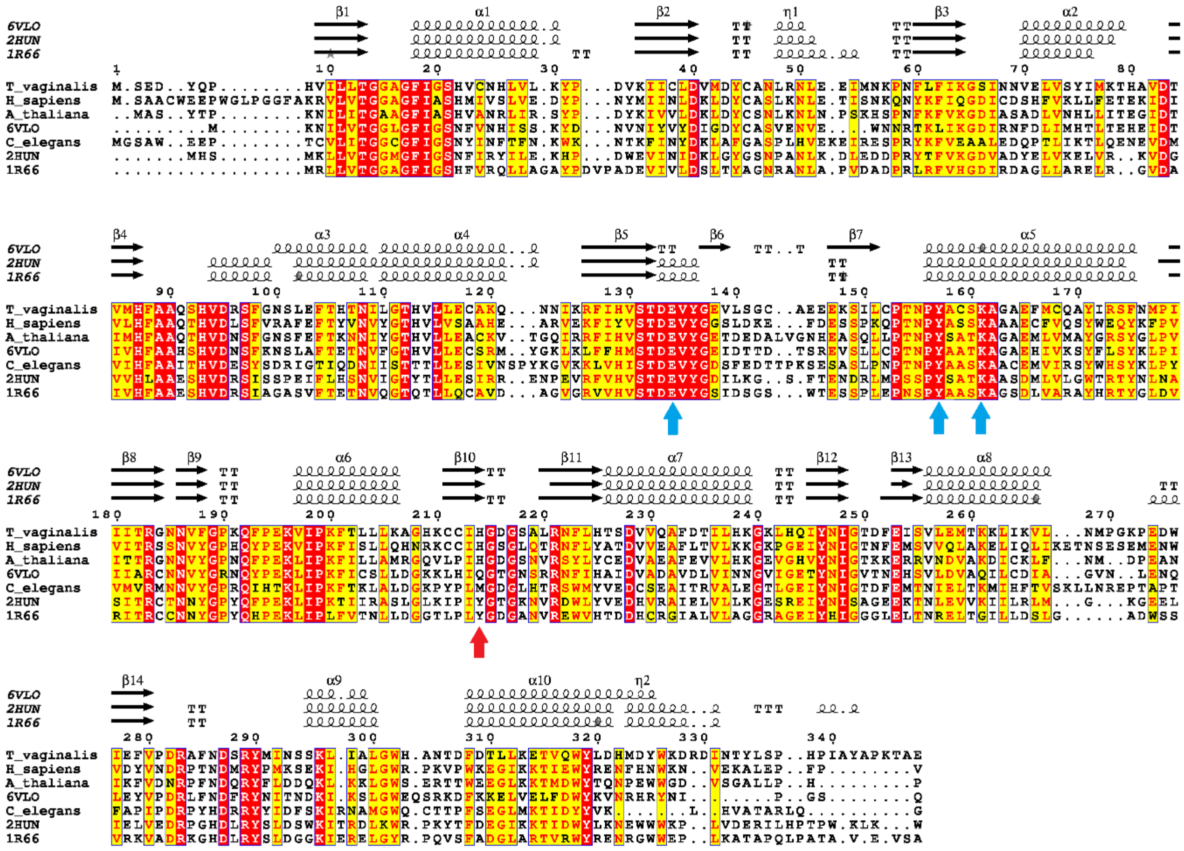

Supplemental Figure S1. Sequence alignments of TvUGD with representative orthologs from different kingdoms. *T. vaginalis*, TVAG_414560; *H. sapiens*, NP_508390; *A. thaliana*, N-terminal domain of RHM1, NP_177978; PDB:6VLO, *Acanthamoeba polyphaga* Mimivirus; *C. elegans* CELE_F53B1.4, NP_055120; PDB:2HUN, *Pyrococcus horikoshii* OT3; PDB:1R66*, Streptomyces venezuelae*. Residues involved in the catalytic mechanisms are indicated by blue arrows. The position corresponding to His214 in TvUGD is highlighted by a red arrow.

Figure S2

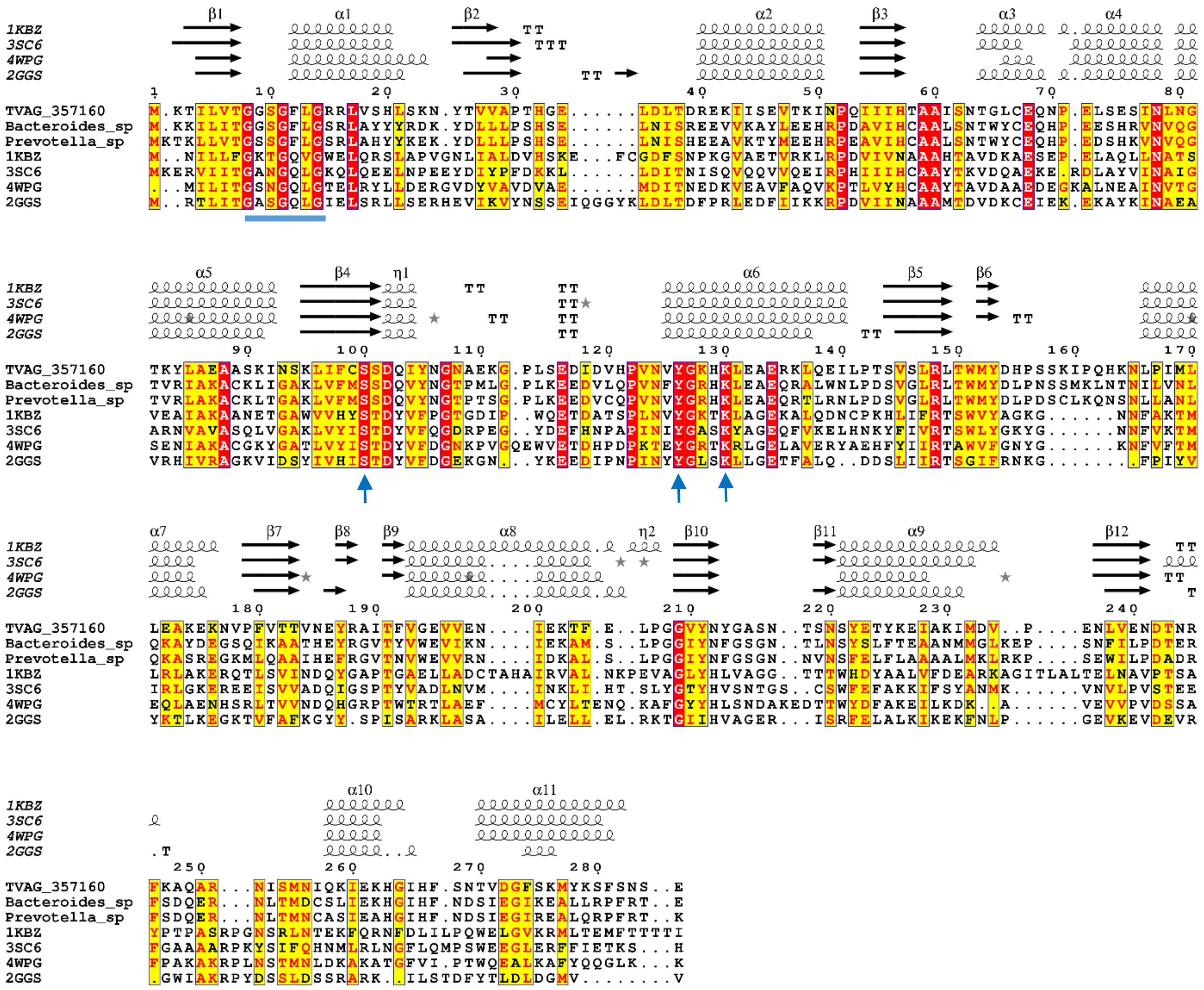

Supplemental Figure S2. Alignment of TVAG_357160 (TvRED) with BLASTp best hits from *Bacteroides* and *Prevotella* species and with characterized bacterial reductases. B*acteroides* sp. CAG:530, CDA77183.1; *Prevotella sp.*, MBD9036083; 1KBZ, *Salmonella typhimurium*; 3SC6, *Bacillus anthracis*; 4WPG, *Streptococcus pyogenes*; 2GGS, *Sulfolobus tokodai*. Residues of the catalytic triad are indicated by blue arrows. The GXXGXXG motif involved in coenzyme binding is indicated by a blue line.

Figure S3

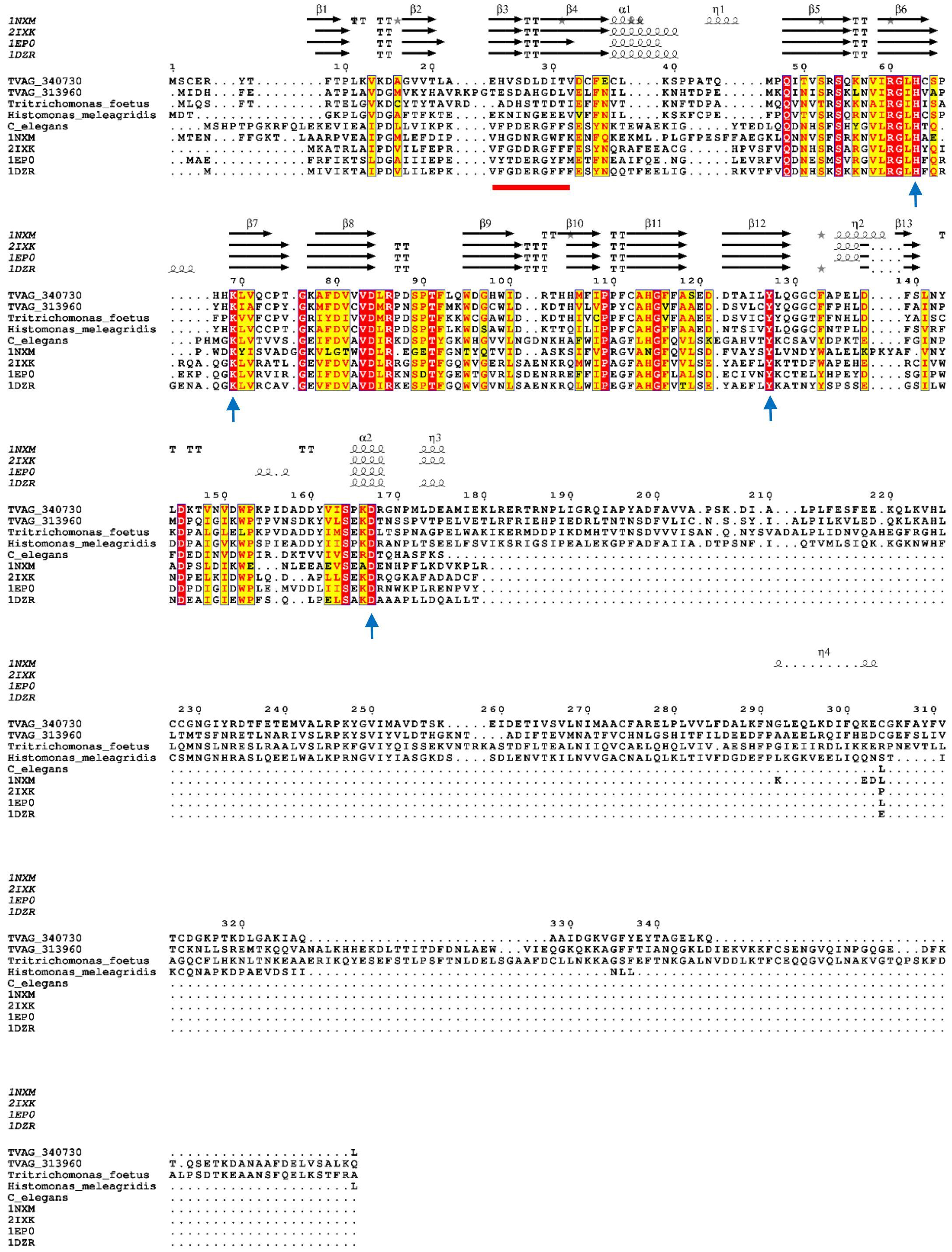

Supplemental Figure S3. Alignment of TVAG_340730 and TVAG_313960 (TvRmlC proteins) with the trichomonad orthologs and with characterized bacterial RlmC 3,5-epimerases. *Tritichomonas foetus* (TRFO_42104, OHT16059); *Histomonas meleagridis* (KAH0792498); *C. elegans* (NP_509046); 1NXM, *Streptococcus suis*; 2IXK, *Pseudomonas aeruginosa*; 1EP0, *Methanothermobacter thermautotrophicus*; 1DZR, *Salmonella typhimurium*. Residues important for catalysis are highlighted by blue arrows. A region involved in dimerization, well conserved in Bacteria and not in trichomonad proteins, is highlighted by a red line.

Figure S4

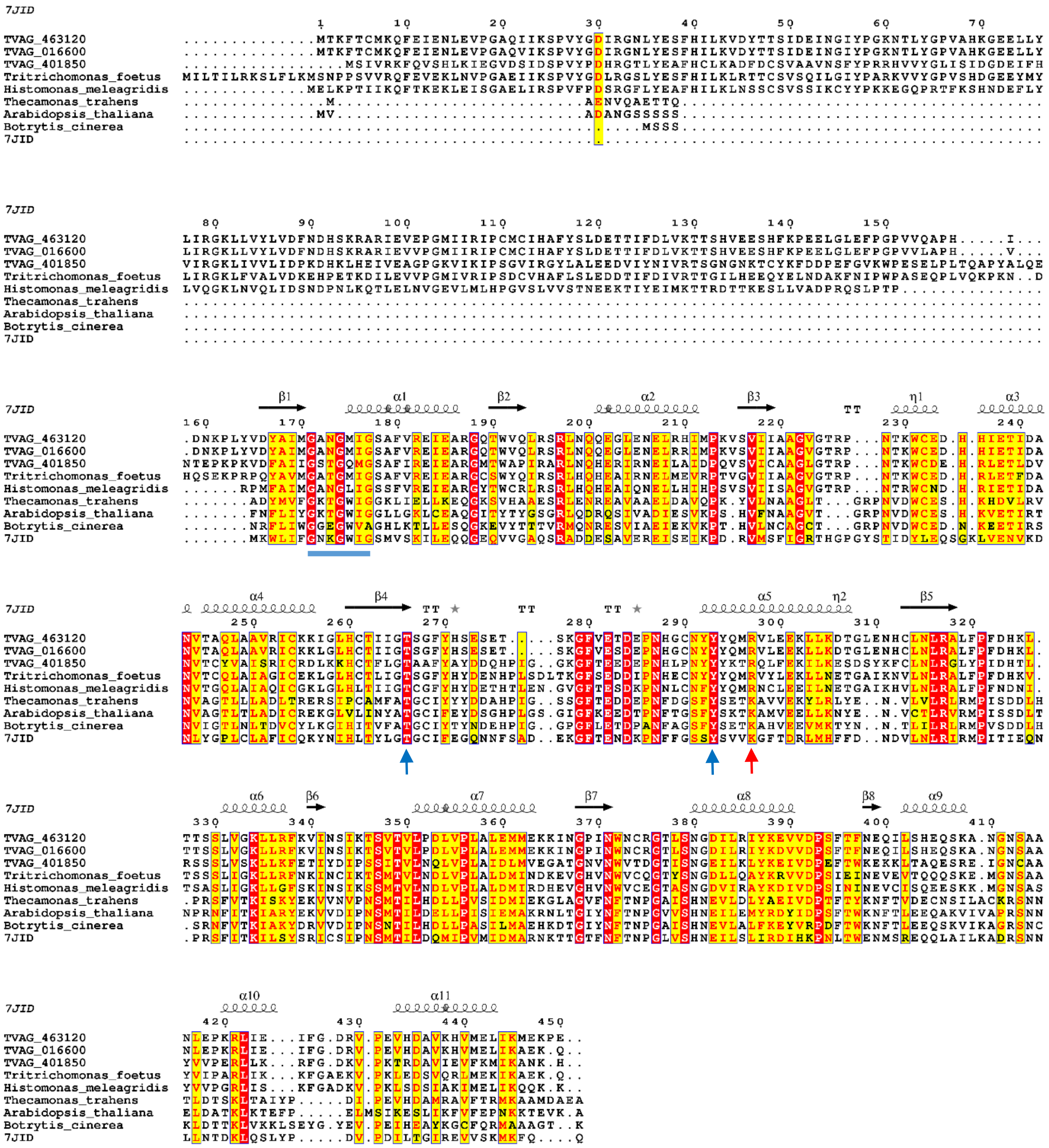

Supplemental Figure S4. Alignment of TvRmlCD proteins with the trichomonad orthologs and the eukaryotic and viral 3,5-epimerase/4-reductase. The GXXGXXG motif involved in coenzyme binding is indicated by a blue line. Residues of the catalytic triad are indicated by arrows; substitution of the well conserved Lys with Arg in trichomonad proteins is highlighted by a red arrow. Accession numbers: *Tritrichomonas foetus*, (TRFO_30760) OHT02199; *Histomonas meleagridis*, KAH0788556; *Thecamonas trahens*, XP_013762229; *Arabidopsis thaliana*, NP_564806; *Botrytis cinerea*, XP_001560439; Mimivirus L780, 7JID.

Figure S5

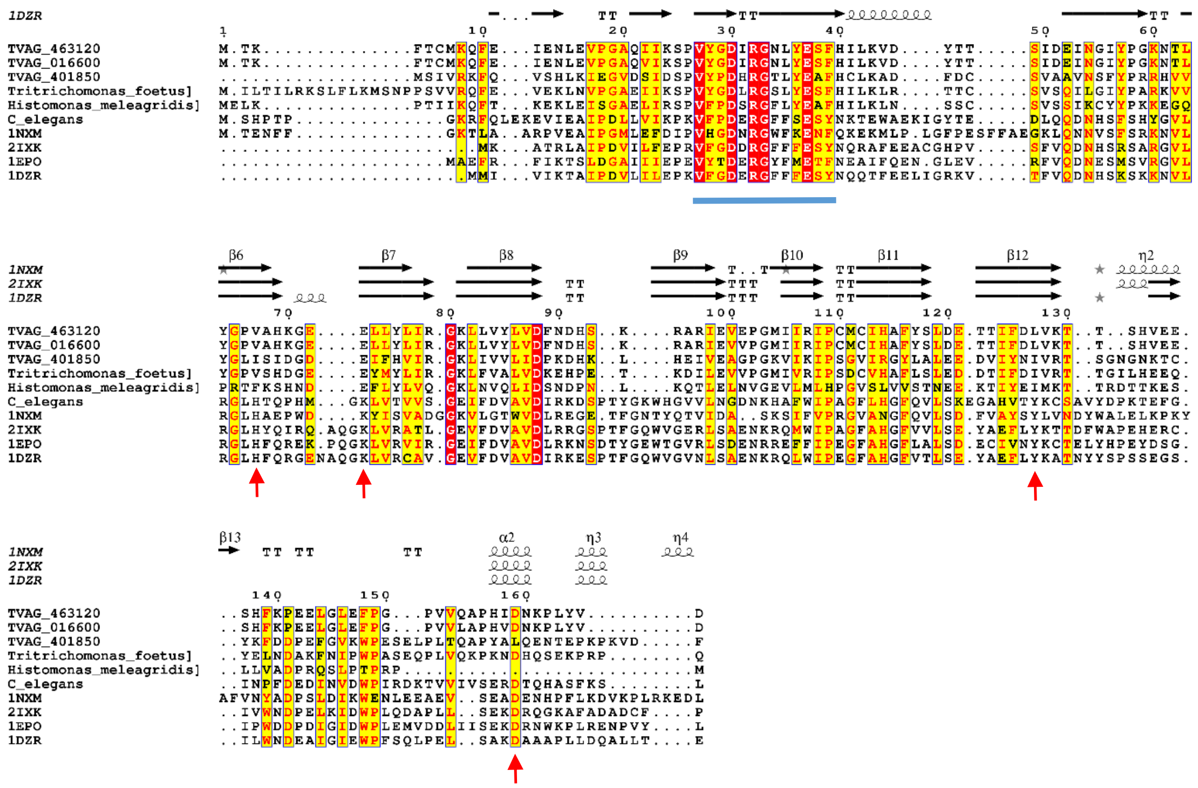

Supplemental Figure S5. Alignment of the cupin-like N-terminal region of TvRmlCD proteins and its trichomonad orthologs with the characterized dTDP-4-dihydrorhamnose 3,5-epimerases from *C. elegans* and Bacteria and. *Tritichomonas foetus*, (TRFO_42104) OHT16059; *Histomonas meleagridis*, KAH0792498; *C. elegans*, NP_509046; 1NXM, *Streptococcus suis*; 2IXK, *Pseudomonas aeruginosa*; 1EP0, *Methanothermobacter thermautotrophicus*; 1DZR, *Salmonella typhimurium*. The prokaryotic sequences are the same reported in Supplemental figure S2. Residues important for catalysis identified in Bacteria, which are not conserved in the trichomonad proteins, are highlighted by red arrows. A region at the N-terminus well conserved in Bacteria, involved in protein dimerization and also conserved in trichomonad proteins is highlighted by a blue line.

Figure S6

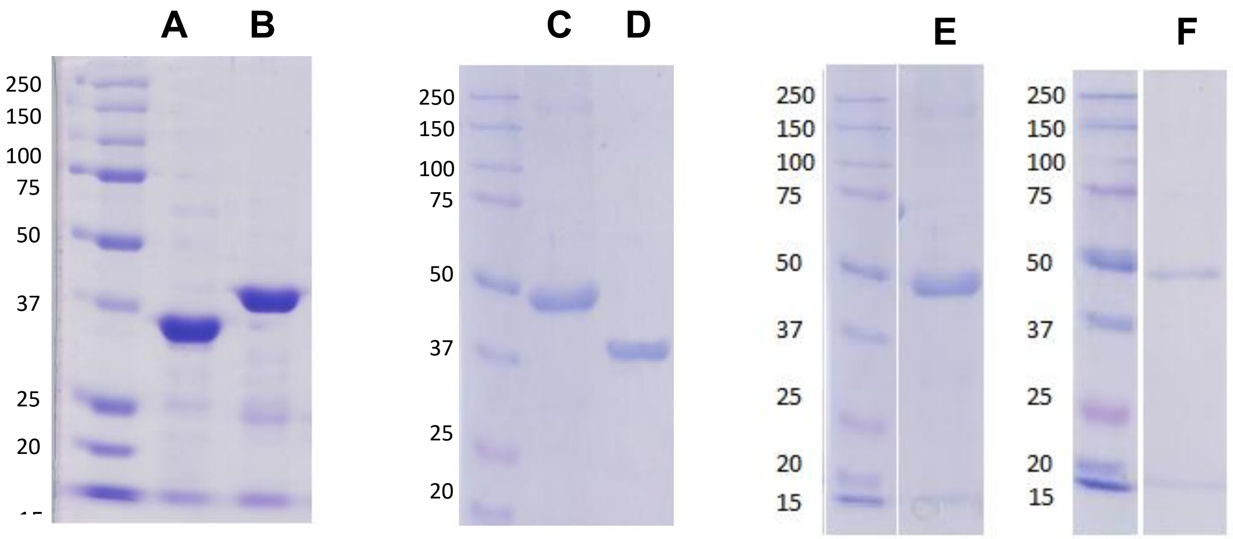

Supplemental Figure 6. SDS-PAGE analysis of the recombinant proteins after expression in *E. coli*, purification and proteolytic cleavage of GST. (A) TVAG_357160, TvRED, predicted mass 31.9 kDa; (B) TVAG_414560, TvUGD, predicted mass 39.5 kDa; (C) TVAG_313960, predicted mass 47,6 kDa; (D) TVAG_340730, predicted mass 38.9 kDa; (E) TVAG_463120, predicted mass 50.9 kDa; (F) TVAG_401850, predicted mass 51.8 kDa.

Figure S7

A

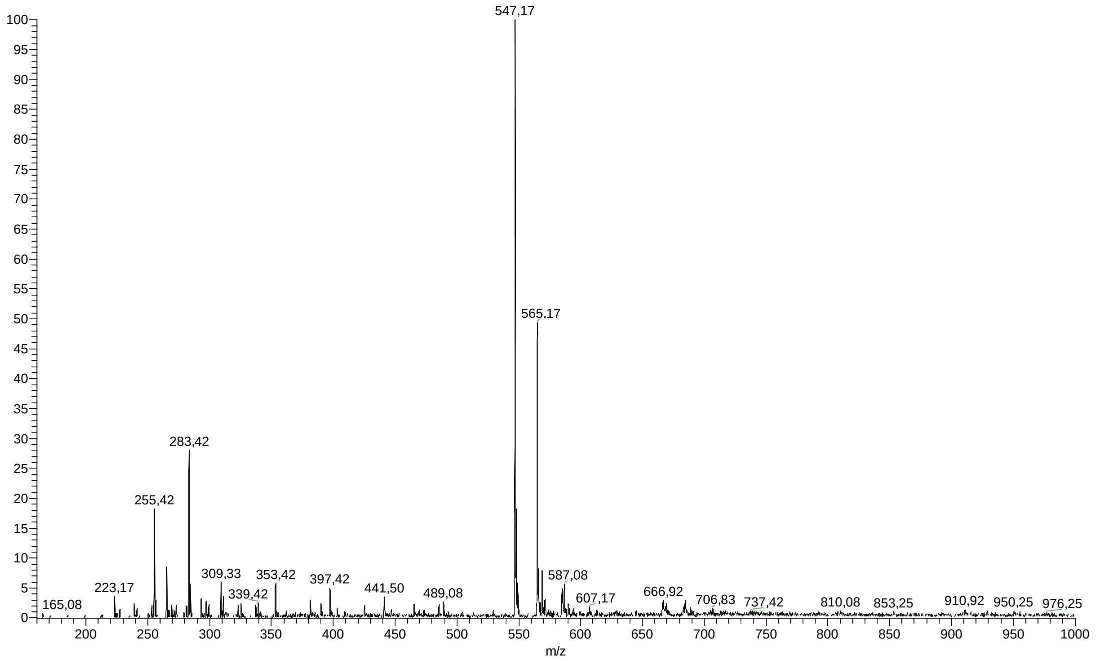

B

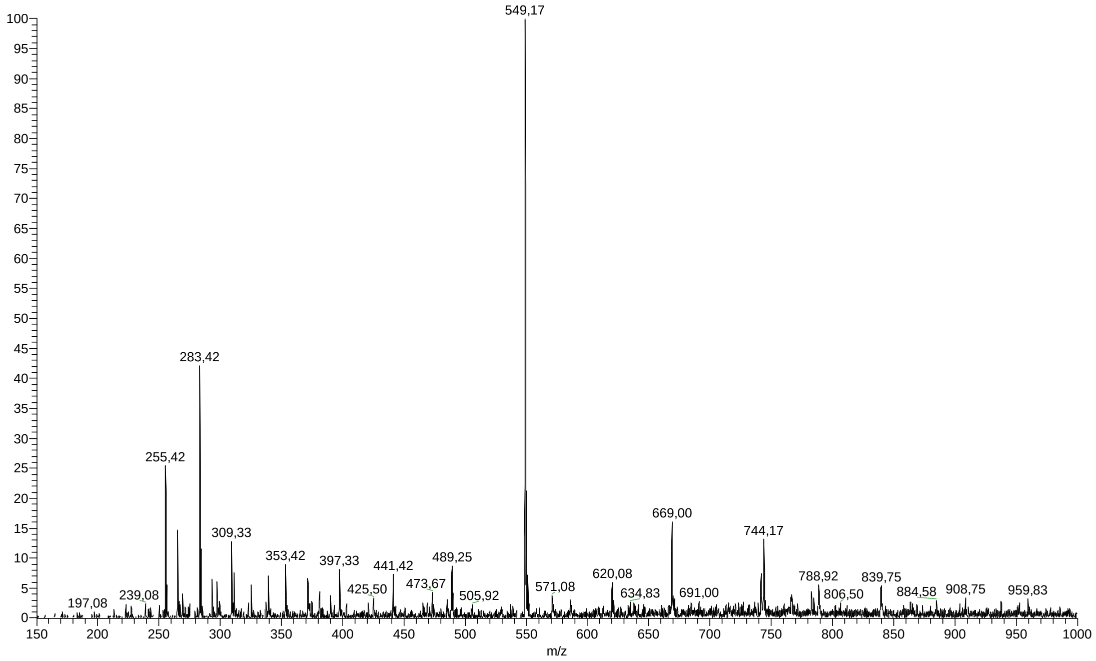

Supplemental Figure S7. ESI-MS analysis of the products. (A) Product obtained after incubation of UDP-D-Glc (m/z 565.17) in the presence of UGD. A new peak at m/z 547.17 corresponds to the dehydration product. (B) Product obtained after incubation of UDP-4-keto-6-deoxy-D-Glc in the presence of NADPH and Tv RED. The peak at m/z 547.17 of the substrate is converted to a new one at m/z 549.17, corresponding to the reduction product of the 4-keto group.

Figure S8

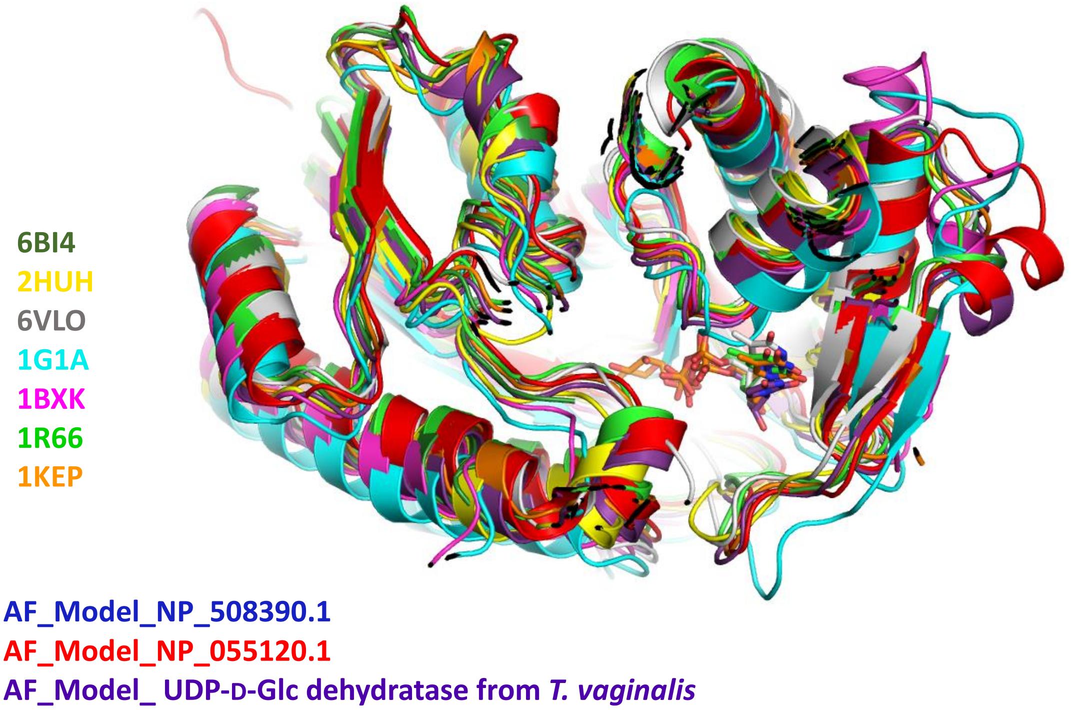

Supplemental Figure S8. 3D alignment of NDP-D-Glc 4,6-dehydratases. *Bacillus anthracis* str. Ames (PDB:6BI4 in dark green), *Pyrococcus horikoshii* OT3 (PDB:2HUN in yellow), *Acanthamoeba polyphaga* mimivirus (PDB:6VLO in grey), *Salmonella enterica* (PDB:1G1A in cyan), *Escherichia coli* (PDB:1BXK in magenta), *Streptomyces venezuelae* (PDB:1R66 in green) (*Streptococcus suis* (PDB:1KEP in orange), *Homo sapiens,* NP_508390 (AlphaFold2 model in blue), CELE_F53B1.4 from *Caenorhabditis elegans,* NP_055120.1 (AlphaFold2 model in red), model of UDP-glucose dehydratase from *Trichomonas vaginalis,* TVAG_414560 (Alphafold2 model, in violet).

Supplemental Table S1. Sequences used for the phylogenetic analysis

XP_004336227	Acanthamoeba castellanii
6VLO_A	Acanthamoeba polyphaga mimivirus R141
YP_001427025	Acanthocystis turfacea chlorella virus 1 (ATCV-1 Z544R)
KYO39463	Alligator mississippiensis
KIH67605	Ancylostoma duodenale
VDK48825	Anisakis simplex
NP_177978	Arabidopsis thaliana (N-terminal domain)
GAQ46732	Aspergillus niger
XP_024547639	Botrytis cinerea
XP_019616331	Branchiostoma belcheri
XP_042931328	Brugia malayi
NP_508390	Caenorhabditis elegans
ELT91052	Capitella teleta
PNW86369	Chlamydomonas reinhardtii
PKK32870	Columba livia
AAH66615	Danio rerio
WP_010889301	Deinococcus radiodurans
XP_005778340	Emiliania huxleyi
XP_652489	Entamoeba histolytica
1BXK_A	Escherichia coli
XP_003545554	Glycine max (N-terminal domain)
XP_005818916	Guillardia theta
WP_042662305	Haloferax volcanii
XP_009031751	Helobdella robusta
NP_055120	Homo sapiens
WP_004032604	Methanobrevibacter smithii
WP_010877391	Methanothermobacter thermautotrophicus
XP_001746636	Monosiga brevicollis
NP_083854	Mus musculus
WP_003418607	Mycobacterium tuberculosis
XP_013307238	Necator americanus
WP_118824249	Neisseria meningitidis
XP_001628519	Nematostella vectensis
WP_014965875	Nitrosopumilus
XP_001422283	Ostreococcus lucimarinus
XP_024365289	Physcomitrium patens (N-terminal domain)
WP_003096179	Pseudomonas
2HUN_A	Pyrococcus horikoshii
1G1A_A	Salmonella enterica
WP_011073075	Shewanella oneidensis
XP_014151261	Sphaeroforma arctica
1R66_A	Streptomyces venezuelae
XP_024509742	Strongyloides ratti
XP_002291084	Thalassiosira pseudonana
MAY24308	Thaumarchaeota archaeon
XP_013761378	Thecamonas trahens

VDM24296	Toxocara canis
XP_001297395	Trichomonas vaginalis
OHT12629	Tritrichomonas foetus
XP_807120	Trypanosoma cruzi
XP_009653544	Verticillium dahliae
XP_002939327	Xenopus tropicalis

Supplemental Table S2. Primers used to amplify Tv cDNA. Restriction sites are underlined

Gene	*Primer*	*Restiction site*	*Sequence 5'→3'*
TVAG_414560	Forward	BamHI	aattggatccatgagtgaggattatcaacc
	Reverse	SalI	aattgtcgacttattcagctgtctttggag
TVAG_357160	Forward	BamHI	aattggatccatgaaaactattcttgtcac
	Reverse	SalI	aattgtcgacttattcagaatttgaaaaag
TVAG_463120	Forward	BamHI	aattggatccatgactaagttcaccctgcat
	Reverse	SalI	aattgtcgacttattctggtttctccatct
TVAG_401850	Forward	BamHI	aattggatccatgagcatagttcgcaaatt
	Reverse	XhoI	aattctcgagttagtgcttgtttgccttga
TVAG_340730	Forward	BamHI	aattggatccatgagctgtgagagatatac
	Reverse	XhoI	aattctcgagttagagttgcttaagttcac
TVAG_313960	Forward	EcoRI	aattgaattcatgattgattgatcatttcga
	Reverse	XhoI	aattctcgagttattgcttaagtgctgaaa
